# Use of a Smartphone App Versus Motivational Interviewing to Increase Walking Distance and Weight Loss in Overweight/Obese Adults With Peripheral Artery Disease: Pilot Randomized Trial

**DOI:** 10.2196/30295

**Published:** 2022-02-03

**Authors:** Tracie Collins, Mugur Geana, Kathryn Overton, Mary Benton, Liuqiang Lu, Faarina Khan, Mason Rohleder, Jasjit Ahluwalia, Ken Resnicow, Yiliang Zhu

**Affiliations:** 1 College of Population Health University of New Mexico Albuquerque, NM United States; 2 School of Journalism and Mass Communications University of Kansas Lawrence, KS United States; 3 University of New Mexico Albuquerque, NM United States; 4 University of Kansas School of Medicine Wichita, KS United States; 5 Brown University Providence, RI United States; 6 University of Michigan Ann Arbor, MI United States; 7 University of New Mexico, School of Medicine Albuquerque, NM United States

**Keywords:** mobile health, smartphone app, peripheral artery disease, motivational interviewing

## Abstract

**Background:**

Walking therapy improves functional outcomes in patients with peripheral artery disease (PAD). Less is known about the additive benefit of a dietary intervention.

**Objective:**

Our objectives were to develop a smartphone app and, as a pilot, explore its potential efficacy as compared to motivational interviewing (MI) to increase walking distance and promote weight loss in overweight/obese adults with PAD.

**Methods:**

We conducted a 3-month, 2-arm randomized pilot study at the University of Kansas. Inclusion criteria were BMI >27 kg/m^2^ and symptomatic PAD, defined by an ankle-brachial index <0.9. Patients were randomized into 2 groups: MI, delivered through in-person and telephone counseling, and app, a mobile smartphone app. Both interventions encouraged walking for exercise and healthy dietary habits (increasing fruits and vegetables and whole grains while reducing fat and sugary drinks). We assessed medical history at baseline. At baseline and 3 months, participants completed an assessment of 6-minute walking distance, weight, quality of life, exercise behaviors, and dietary habits. The primary outcome was 3-month change in walking distance. Secondary outcomes were changes in weight, quality of life, exercise behaviors, and dietary habits. We used a Wilcoxon rank-sum test to analyze the primary and secondary outcomes at 3 months within the MI and app groups and to compare the changes between the groups with adjustment for baseline.

**Results:**

We randomized 29 participants with a mean age of 66.03 (SD 8.12) years; 25 participants completed the trial. At baseline, mean walking distance among completers was 260.40 (SD 94.32) meters and 326.15 (SD 69.28) meters for MI and app participants, respectively. At 3 months, the mean walking distance was 298.67 (SD 101.20) meters and 331.19 (SD 58.63) meters for MI and app participants, respectively (group difference *P*=.03, adjusting for baseline). Increase in walking distance at 3 months was 40.5 meters (95% CI 6.77 to 61.34; *P*=.02) in MI group. At baseline, mean body weight was 253.10 (SD 59.45) lbs and 225.13 (SD 58.93) lbs for MI and app participants, respectively. At 3 months, mean body weight was 242.14 (SD 58.54) lbs and 223.44 (SD 59.54) lbs for MI and app, respectively (group difference *P*=.006, adjusting for baseline). Pre-post study decrease in weight was 10.1 lbs (95% CI –17.9 to –3.0) and 2.3 lbs (95% CI –3.4 to –0.7) in MI and app group, respectively. Comparing baseline to 3 months, there were no statistically significant differences in quality of life, exercise behaviors, or dietary habits.

**Conclusions:**

Our study demonstrates that MI can promote walking and weight loss in overweight/obese adults with PAD. The smartphone app showed a small weight loss but no statistically significant increase in walking distance. As this was a pilot study, future large-scale studies are needed to replicate the efficacy of MI to promote weight loss in overweight or obese adults with PAD.

**Trial Registration:**

ClinicalTrials.gov NCT03694652; https://clinicaltrials.gov/ct2/show/NCT03694652

## Introduction

Peripheral artery disease (PAD) is atherosclerosis of the abdominal aorta and arteries of the lower extremities that causes stenosis or occlusion [1]. Patients with PAD are at increased risk for cardiovascular events and functional decline [2-5]. As such, treatment for PAD has focused not only on risk factor modification but also on improving walking distance [6-10].

Community-based walking therapy is an effective noninvasive treatment option for PAD [11-13]. Motivating adherence to community-based walking therapy can be achieved through traditional, in-person approaches. Alternatively, motivating adherence can be achieved through mobile health or counseling [8,9]. We explored the potential efficacy of a smartphone app versus a well-known counseling approach, motivational interviewing (MI), to improve walking distance and promote weight loss among overweight/obese adults with PAD.

Although walking therapy is an important component of improving outcomes in patients with PAD, less is known about the efficacy of dietary interventions for disease management—in particular, to promote weight loss in overweight/obese patients with PAD.

Our pilot study is novel insofar as it focuses on walking therapy combined with dietary intervention. We delivered this combined intervention using 2 approaches: a smartphone app versus MI, delivered both in person and by phone. The app offers an innovative approach to behavior change for patients with PAD, and the use of MI builds upon our prior work involving patients with PAD [14,15]. The app was developed by MG and his team in the School of Journalism and Mass Communications, University of Kansas, Lawrence, Kansas. The theoretical framework for the app includes the transtheoretical model, social cognitive theory, and self-determination theory. Similarly, for MI, the theoretical framework includes the self-determination theory and transtheoretical model. In addition, MI is based on client-centered therapy [16-19].

In this pilot, we explored the potential efficacy of our app versus MI to increase walking distance and promote weight loss among overweight/obese adults with PAD.

## Methods

### Participants

We conducted a 2-arm, pilot randomized trial. Inclusion criteria were age 50 years or older, overweight/obese (BMI >27), and symptomatic PAD—leg symptoms were captured by a survey, and PAD was confirmed with the use of the ankle-brachial index, the ratio of the systolic blood pressure in the ankle to that in the arm. Our cut point for the ankle-brachial index was <0.9.

Individuals were excluded if they demonstrated at least one of the following conditions: intolerance to fruits, vegetables, fiber, or a low-fat diet; restricted water intake; pregnancy; prior major ischemia or critical leg ischemia; use of 24-hour supplemental oxygen; heart attack within the last 3 months; inability to walk for exercise; or currently walking at least 3 days per week for at least 30 minutes each day. The University of Kansas Medical Center institutional review board approved this study, and participants gave informed consent. This clinical trial was registered at ClinicalTrials.gov [NCT03694652].

### Recruitment

We recruited study participants using a variety of modalities including the University of Kansas hospital system and unaffiliated health centers and community-based clinics; area clinicians and administrators voluntarily distributed flyers and mailings to eligible patients. We obtained university institutional review board approval and permission from participating hospitals and clinics prior to each phase of the recruitment process.

### Interventions

#### Motivational Interviewing

Participants randomized to MI participated in an initial 1-hour, face-to-face session. Following the initial visit, we conducted four 20-minute telephone calls: 1 call every 2 weeks for 1 month followed by 2 monthly phone calls.

Author KR provided MI counselor training through a series of workshops. The counselor was a medical school graduate and a student in the master’s in public health program at University of Kansas School of Medicine–Wichita who was also applying for residency in primary care. Although the counselor had no experience in MI prior to this trial, author KR has trained multiple research staff in the delivery of MI. There were standardized patients and ongoing review of sessions through direct observation. The counselor’s goals were to elicit and reinforce change talk about increasing walking behavior and healthy dietary habits (Table 1).

During each counseling session, the counselor assessed participant willingness to walk for exercise using the Patient-Centered Assessment and Counseling for Exercise (PACE) survey, which assesses readiness to exercise [20,21]. Following a discussion of their current PACE score, the counselor queried patients about the importance of exercising and their current motivation to increase walking behavior. The counselor concluded each session by offering patients the option to set a walking goal for the next 2 weeks.

The counselor followed a similar process with respect to the nutritional portion of the interview. She guided patients to choose 1 of 6 nutritional options to work on in the following 2 weeks: (1) reducing sodium, (2) increasing fruits, (3) increasing vegetables, (4) decreasing sugary drinks, (5) increasing whole grains, or (6) mindful eating. Similar to the exercise therapy, the counselor discussed the importance of change, patient confidence levels, and individual values. After patients established exercise and nutritional goals, counselors would arrange for subsequent follow-up sessions at 2-week intervals.

**Table 1:** Behavior change techniques: nutrition and physical activity.

Technique	MI^a^	App
1.1 Goal setting (behavior): agreed on weekly walking goals	✓	✓
1.2 Problem solving: identifying triggers to eating unhealthy foods or avoiding walking for exercise	✓	✓
1.4 Action planning: setting aside time to exercise and planning meals in advance	✓	✓
1.6 Discrepancy between current behavior and goal: recorded walking goals or self-reported dietary goals were not met	—^b^	✓
3.1 Social support (unspecified): participants received recommendations on the value of having a buddy to walk with	✓	✓
4.1 Instruction on walking therapy to improve walking distance	✓	✓
4.3 Re-attribution: if a participant attributed their desire for food to boredom, we provided guidance on mindful eating	—	✓
5.1 Information about health consequences: participants were provided information about the potential for disease progression in the absence of a walking intervention	✓	✓
5.4 Monitoring of emotional consequences: participants were queried about satisfaction with their weekly dietary and walking goals	—	✓
8.2 Behavior substitution: participants were provided with guidance on substituting unhealthy dietary choices with healthy dietary choices	—	✓
9.1 Credible source: participants viewed videos in which the principal investigator, who is board-certified in internal and vascular medicine, shared the importance of walking for exercise for persons with peripheral artery disease	—	✓
9.2 Pros and cons: participants were queried regarding the pros and cons of eating a healthy diet and walking for exercise	✓	✓
10.4 Social reward: participants were congratulated for achieving their weekly goals	—	✓
13.2 Framing/reframing: participants were provided with cognitive structuring to think of tasks to reduce sedentary behavior	✓	✓
15.1 Verbal persuasion about capability: participants were told they can walk for exercise despite leg discomfort	✓	✓

^a^MI: motivational interviewing.

^b^Not applicable.

#### Smartphone App

##### Development

We created the PAD mobile app with support from the Center for Excellence in Health Communication to Underserved Populations at the University of Kansas School of Journalism and Mass Communication. Author MG supervised the design, operational functionality, and development of the app. We employed iterative feedback from experts and research team members to design the user interface, content, and functionality.

The app was designed for use with Android OS smartphones (patients without this type of phone received it as part of the study). We tailored nutritional content for clarity and lower health literacy levels. We designed the mobile app to allow users to input walking plans, track walking intervals, and record episodic pain. We designed in-app notifications to encourage patient use and for diet and exercise management.

The app was completely automated and adaptive to patient use. The study team was available to troubleshoot and answer questions as needed. Safeguards permitted recovery of previously saved data in the event of accidental malfunction. Participant data were encrypted and saved locally to the phone. Once initialized, the app sent secure, depersonalized data to an online backup server via the internet.

##### Implementation

We developed the PAD mobile app specifically for this pilot study based on the PACE and MI principles described previously. The PAD mobile app had 2 distinct components: a nutritional component and a walking component. Similar to the MI group content, the nutritional component of the mobile app contained 6 modules, with each of the topics designed to be completed over a 2-week period. We designed each module to reflect nutritional issues specific to PAD patients. Patients were permitted to choose the order the modules for completion, and completion was self-initiated. The app employed identical measures for assessing patient perceptions of the importance of change, confidence levels, and individual values. The app used the PACE format to set exercise goals, and self-assessment to set nutritional goals over a 12-week period. Similar to the MI group content, nutritional modules were further divided into six 2-week cycles.

On the first day of the study, patients installed the app, and it prompted them to enter their demographic, health, and identity information. The app then presented patients with 6 possible nutritional changes that they could choose to address during the first 2-week cycle. Once participants chose a dietary modification, they were prompted to perform a self-assessment; the app would then adjust goals according to patients’ responses.

**Figure 1:**
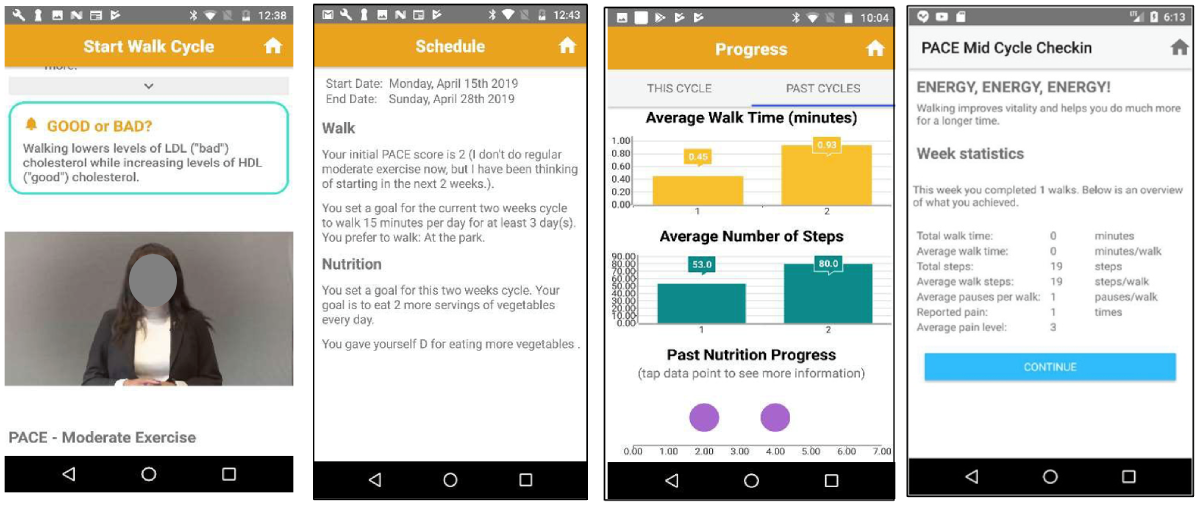
PAD mobile app: components of walking and nutrition modules.

We made explicit efforts to ensure that patients would not have trouble installing, using, or interacting with the app. Several safeguards were installed to provide patients with continuous support and resources. The app provided extensive exercise tracking (Figure 1) as well as a comprehensive reporting menu with information about patient progress (Figure 1). Automatic reminders prompted participants to engage with the app and to select their next PAD intervention cycle at 2-week intervals.

### Measures

#### Ankle-Brachial Index

The ankle-brachial index was used to define the presence or absence of PAD. During this assessment, a participant rested for 5 minutes after which a 5 MHz hand-held Doppler with an attached stethoscope was used to measure systolic blood pressures in both brachial arteries and in both ankles (ie, the dorsalis pedis and posterior tibial arteries) [22].

#### Medical History

The principal investigator and colleagues developed the Lifestyle and Clinical Survey (Multimedia Appendix 1) to obtain patients’ medical history, including smoking status and sociodemographic and comorbidity data. It has a summary k-statistic for reliability of 0.81 (95% CI 0.78 to 0.84) and a summary k-statistic for validity of 0.58 (95% CI 0.52 to 0.64) [23].

#### Stage of Readiness to Engage in Exercise

The PACE score was used to identify a participant’s readiness for exercise. To obtain a PACE score, a participant chose 1 of 8 graded statements that best described their current level of and interest in physical exercise [24]. We assessed a participant’s PACE score at baseline and 6 weeks; we employed these assessments to tailor the intervention to each patient’s respective stage of readiness.

### Outcomes

The primary outcome was the 3-month change in walking distance as measured by the 6-minute walk test, which provides information on a patient’s ability to walk in the community. The test is conducted by placing 2 cones 100 feet apart in a marked hallway and instructing patients to walk as many laps around the cones as possible. Patients were permitted to stop walking during the test; however, time was recorded during the rest period. We recorded time and distance to onset of leg discomfort and total distance walked. In a prior study involving 64 patients with PAD, the reliability coefficient for distance during 6-minute walk tests performed 1 week apart was 0.94 with a coefficient of variation of 11.7% [25].

Secondary outcomes included weight loss, quality of life, exercise behaviors, and dietary habits. For weight loss, we used a standardized scale to measure body weight. For health-related quality of life, we used the Vascular Quality of Life Questionnaire (VascuQoL) [26]. The intraclass (reliability) coefficient for the VascuQoL was 0.94 (CI >90%) and Cronbach α ranged between .7 and .9, indicating good internal consistency within the 5 domains. The VascuQoL total score has a correlation with the Fontaine classification of disease severity [27] of *r*=–.79 (*P*<.001) and with treadmill walking distances of *r*=.36 (*P*<.05). For self-reported physical activity, we used the Stanford Patient Education Research Center Exercise Behavior Survey [28,29]. The Exercise Behavior Survey retest reliability scores between 0.56-0.72. We employed the Fat-Related Diet Habits Questionnaire [30] to assess basic consumption as well as healthier choices, such as substitution of low-calorie or low-fat food alternatives. We modified the survey to a 3.9 reading level. The Fat-Related Diet Habits Questionnaire has high test-retest and internal consistency reliabilities and correlations with percentage of calories from fat ranging from 0.34 to 0.57 (*P*<.01). The correlation of the sum of the 5 scales with percentage of calories from fat was 0.68 (*P*<.001) and, in multiple regression models, the multiple *R*^2^ using all factors to predict percentage of calories from fat was 0.47. All outcomes were measured at baseline and 3 months.

### Study Flow

#### Screening

We prescreened potential participants via phone. The prescreen assessment captured (1) physical activity readiness (Physical Activity Readiness Questionnaire) to identify conditions that would preclude participation in the study [31] and (2) leg symptoms (San Diego Claudication Questionnaire) [32]. Based on eligibility criteria, we scheduled candidates for an in-person visit.

#### Baseline In-Person Visit and Randomization

After obtaining informed consent, a blinded assessor measured individuals’ ankle-brachial index, weight, and height. We referred eligible candidates to graded submaximal treadmill testing, as per American College of Cardiology guidelines.

Upon completion of treadmill testing and without evidence of coronary ischemia, participants completed questionnaires to assess quality of life, exercise behaviors, and dietary habits. We then randomly assigned participants to the app or MI. We used a random number generator to blindly assign patients to one of the 2 groups.

#### Analysis Plan

We used a Wilcoxon rank-sum test to analyze the primary outcome of change in 6-minute walking distance at 3 months within the MI and app groups and compare the changes between the groups with adjustment for baseline (difference in difference). We used this same method for other outcomes. For comparing participants’ baseline characteristics between the MI and app groups, we used a Wilcoxon test for continuous measurements and a Fisher test for dichotomous measurements. We use R (version 4.03, R Foundation for Statistical Computing) for all data analyses.

Thirteen participants per group were required to provide 80% power at a 0.05 error level and to detect a difference between the 2 groups of 50 meters walking distance change.

## Results

**Figure 2:**
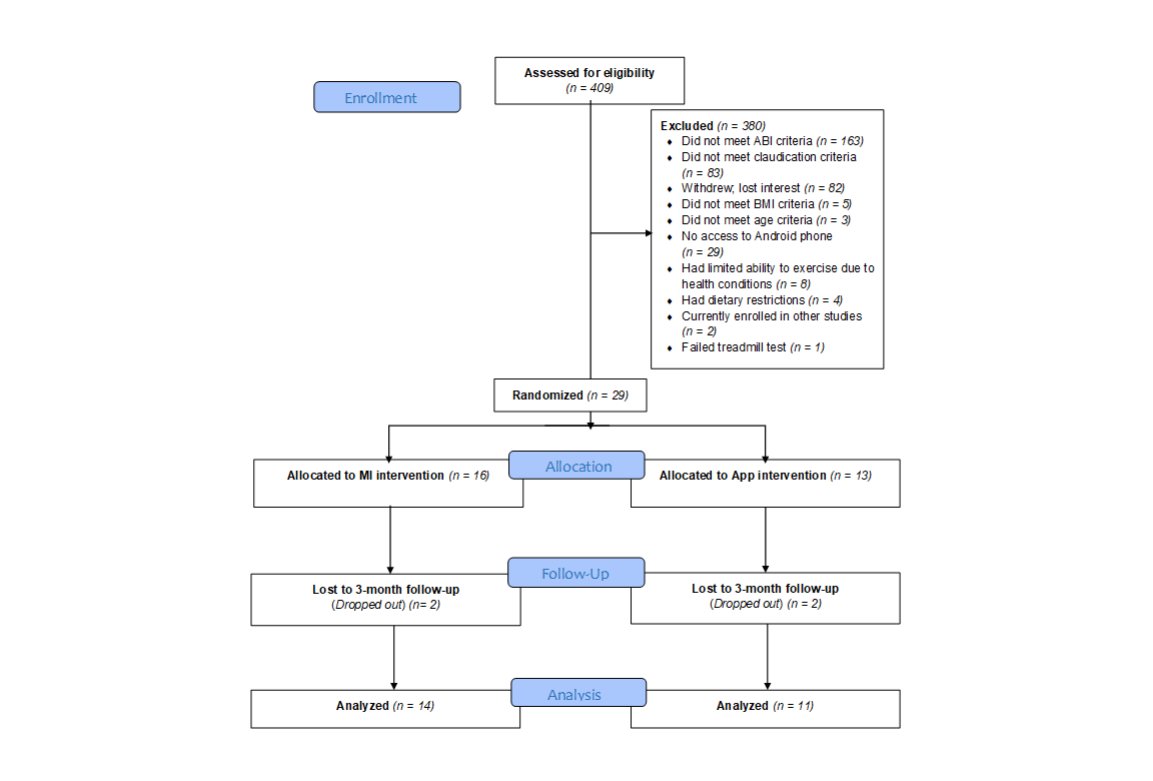
CONSORT flow diagram. ABI: ankle-brachial index; MI: motivational interviewing

We randomized a total of 29 participants and assigned 16 to MI and 13 to the app (Figure 2); 25 participants completed the 3-month assessment (MI=14 and app=11), which retained >75% power.

Baseline characteristics of all 29 participants are shown for the entire sample and by treatment group in Table 2. For the overall cohort, the mean age was 66.0 (SD 8.12) years. There was no group difference in prevalence of cardiovascular risk factors or alcohol use.

At baseline, mean walking distance by group was 260.40 (SD 94.32) meters for MI and 326.15 (SD 69.28) meters for the app. At 3 months, the mean walking distance was 298.67 (SD 101.20) meters for MI and 331.19 (SD 58.63) meters for the app. The median increase in walking distance was 40.51 meters (95% CI 6.77 to 61.34, *P*=.02) in MI and 8.04 meters (95% CI –15.86 to 22.92; *P*=.41) in app. Group difference in change of walking distance was 33.60 meters (95% CI 8.69 to 58.39, *P*=.03).

At baseline, mean body weight by group was 253.10 (SD 59.45) lbs for MI and 225.13 (SD 59.83) lbs for the app. At 3 months, mean body weight by group was 242.14 (SD 58.54) lbs for MI and 223.44 (SD 59.54) lbs for the app. The median weight decrease was 10.1 lbs (95% CI –17.9 to –3.0; *P*=.01) in MI and 2.3 lbs (95% CI –3.4 to –0.7; *P*=.02) in app. Group difference in weight change was –7.48 lbs (95% CI –14.60 to –3.20; *P*=.006).

**Table 2:** Baseline participants characteristics.

Characteristics		Overall (n=29)	MI^a^ (n=16)	App (n=13)	*P* value
Age (years), mean (SD)		66.03 (8.12)	68.39 (7.36)	63.12 (8.34)	.01
BMI, mean (SD)		38.25 (9.25)	39.69 (10.18)	36.47 (8.00)	.37
Weight (lbs), mean (SD)		236.67 (59.05)	244.30 (60.38)	227.28 (58.36)	.53
Waist, mean (SD)		48.12 (5.78)	48.41 (6.18)	47.77 (5.49)	.63
Income ($), mean (SD)		50,805.43 (39,571.16)	50,534.5 (47,581.8)	51,166.67 (30,022.77)	.59
Female, n (%)		20 (70)	11 (69)	9 (69)	>.99
Educations (≥ high school), n (%)		28 (97)	16 (100)	12 (92)	.45
Worried about housing loss, n (%)		3 (10)	1 (6)	2 (15)	.57
**Employment, n (%)**					
	Full-time work	12 (41)	5 (31)	7 (54)	.22
	Unemployed but not seeking work	15 (52)	10 (63)	5 (38)	.27
**Insurance, n (%)**					
	None/uninsured	4 (14)	2 (13)	2 (15)	>.99
	Medicare	16 (55)	11 (69)	5 (38)	.14
	Private insurance	8 (28)	3 (19)	5 (38)	.41
**Health history, n (%)**					
	Myocardial infarction	8 (28)	4 (25)	4 (31)	>.99
	Cardiac catheterization	12 (41)	9 (56)	3 (23)	.13
	Claudication	9 (31)	5 (31)	4 (31)	>.99
	Hypertension	23 (79)	12 (75)	11 (85)	.66
	Hypercholesterolemia	19 (68)	10 (63)	9 (69)	>.99
	Diabetes	13 (45)	8 (50)	5 (38)	.71
	Diabetes-related complications	8 (28)	7 (44)	1 (8)	.04
	Arthritis other than rheumatoid	12 (41)	9 (56)	3 (23)	.13
	At least 100 cigarettes during lifetime	18 (62)	10 (63)	8 (62)	>.99
	Ethanol use	18 (62)	8 (50)	10 (77)	.25

^a^MI: motivational interviewing.

In addition to walking distance and weight loss, we also assessed changes in quality of life, exercise behaviors, and dietary habits (Multimedia Appendix 2). Overall, there were no differences between the app and MI groups in quality of life, exercise behaviors, and dietary habits, comparing baseline to 3 months.

To encourage patients to complete the nutritional components of the app, researchers sent out reminders both midcycle and at the end of each cycle. Despite these nudges, patient participation with the nutritional component of the app was highly variable. Out of 14 participants, only 3 individuals started all 6 nutrition modules. Among patients randomized to the PAD mobile app, 29% completed all 6 modules, 24% completed 5 modules, and 5% completed only 1 module. A total of 72% (10/14) of participants started 4 nutrition modules or more. Overall, there was a high degree of variability in terms of engagement with the nutritional component of the app.

Included with the nutritional component of the app were guided assessments designed to measure patients’ confidence and personal importance in achieving nutritional goals. Across all of the cycles, patients’ initial average confidence in achieving nutritional goals was 6.4 on a scale from 1 (lowest confidence) to 10 (highest confidence). In addition, the average perceived personal importance of the nutrition topics was 6.5 on a scale from 1 to 10.

The app was designed to prompt self-evaluation of progress at 2 time points during each nutritional module. We designed interactions with the app to mimic the dialogue that occurred at 2-week intervals in the MI group. The use of the app varied across the group of participants, with some using it as little as 8 times during the 12-week period and others using it over 200 times during the same interval. There were a total of 1137 interactions with the app, of which 111 were generated by directly responding to a prompt from an app notification.

Patients entered midcycle data in only 13 out of the 52 started modules (25%). Patients entered end-of-cycle data in only 18 out of the 52 modules initiated. Thus, there was a huge attrition rate associated with participation in the nutritional component of the app. This attrition occurred despite the presence of multiple, automatic system notifications—9, on average—sent to support nutritional goals and to remind participants to log in and enter feedback.

Participants demonstrated substantially greater interaction with the walking component of the PAD mobile app than the nutritional modules. The app tracked a total of 355 walking episodes over the duration of the study; this represented an average of 25 walking episodes per participant.

Patients reported variable levels of pain throughout the 12-week study period. A total of 55% (6/11) of participants recorded less pain on their last cycle compared with the first one. A total of 36% (4/11) recorded more intense pain at their last recorded cycle in comparison to their first.

The app recorded average walking time per recorded walking episode as 00:17:57. Based on limited data from 3 devices, we found that patients walked at an average pace of 2.15 seconds per step. Thus, participants averaged approximately 500 steps per session.

## Discussion

### Principal Findings

We found that MI but not the app promoted increased walking distance and both interventions promoted somewhat significant weight loss among overweight or obese adults with PAD, although the loss in the app group was rather small in size. To our knowledge, this is the first study to demonstrate the potential efficacy of MI to promote weight loss in overweight or obese patients with PAD. Applications of MI to improve outcomes have been demonstrated in prior work. Cunningham and colleagues [33] found that MI was successful in motivating PAD patients to increase walking distances. This pilot confirmed that MI was efficacious to improve walking distance. However, in prior work by Collins et al [15] involving African Americans with PAD, MI was not efficacious. Findings from that study highlight the need among some populations for more physician-directed counseling to motivate adherence to walking therapy. However, understanding the potential efficacy of MI on a larger scale is still warranted.

MI was more efficacious than our app for improving weight loss. The reason for the efficacy of MI versus our app is likely multifactorial. One consideration is the human contact that is afforded by MI as compared to the app (or mHealth). Both human contact and mHealth have their advantages and disadvantages, as noted by Santarossa et al [34]. mHealth should create accountability and social support to successfully motivate behavior change. Although we designed our app to provide support, we can certainly address this more in future work. The theoretical framework underlying MI includes the self-determination theory and transtheoretical model. Thus, the potential mechanisms by which MI was efficacious include intrinsic and extrinsic motivation as well as stages of change. However, there were no statistically significant improvements in dietary habits. A larger study with assessment of potential mediators and moderators for intervention efficacy could shed more light.

Also, some technological design challenges may explain our limited findings with respect to walking distance and weight loss among patients randomized to the app. For example, the current version of the app requires that patients deliberately initiate it during every use. In addition to technological challenges, we observed that patients’ overall use of the mobile app was highly variable. This finding could be improved through additional in-app notifications.

Despite midcycle and end-of-cycle notifications, patient participation with the nutritional component of the app was also highly variable. Among the participants randomized to the app, very few started all 6 nutrition modules. We speculate that complexity of the nutritional components of the app may have contributed to decreased participation. This decreased participation may have in turn contributed to the modest findings among individuals randomized to the app.

### Limitations

The small sample of participants is an obvious limitation of the study as it precludes generalizability and has limited power. We readily acknowledge these constraints as we prepare to move forward with a larger clinical trial in the future.

Additionally, because of licensing constraints imposed by the Apple Corporation, we designed the mobile app for use with Android phones (an Apple version is in development). Relaxing these constraints in the future will allow for a more streamlined, feature-rich, and automated app. Our ultimate goal is to expand accessibility of the app as an mHealth alternative among the broader PAD population. We did not complete in-depth interviews with app participants regarding reasons for or against use of the app. Finally, improvements are needed in the app to increase use. Again, another complication associated with the app is that patients were prompted to provide progress data, but these data needed to be entered voluntarily into the app in order to chart progress. The app was designed to prompt midcycle for an initial evaluation of the progress as well as at the end of the module. The purpose of these data points was to assess and record completion, estimate goal achievement, and identify barriers. Researchers designed interactions with the mobile app to mimic the dialogue that occurred over the phone in the first arm of the study. Thus, the notifications were intended to be similar to conversations that a trained evaluator would have with the patient by telephone every 2 weeks.

### Conclusion

MI significantly improved walking distance and promoted weight loss for participants randomized to MI versus the app. As the use of mobile devices is increasing, the need for development of mobile apps to promote walking therapy and health dietary habits cannot be ignored. The potential of MI to promote weight loss in overweight/obese adults with PAD warrants further study. Additionally, the development of a mobile app that is compatible with IOS as well as Android phones with content available in multiple languages will be the focus of a future study. This pilot project represents one of the first pilot studies to include behavioral interventions targeting both exercise and diet in overweight/obese adults with PAD.

## Appendix

Multimedia Appendix 1 Lifestyle and clinical survey.

Multimedia Appendix 2 Study outcomes by intervention groups for completers only.

Multimedia Appendix 3 CONSORT-eHEALTH checklist (V 1.6.2).

## Abbreviations

MI: motivational interviewing
PACE: Patient-Centered Assessment and Counseling for Exercise
PAD: peripheral artery disease
VascuQoL: Vascular Quality of Life Questionnaire
