# Supplementary material for: Use of a Smartphone App Versus Motivational Interviewing to Increase Walking Distance and Weight Loss in Overweight/Obese Adults With Peripheral Artery Disease: Pilot Randomized Trial
Source: JMIR Form Res. 2022 Feb 3;6(2):e30295. doi: 10.2196/30295 (PMC8855281; doi:10.2196/30295)
Supplement: Multimedia Appendix 1 [file formative_v6i2e30295_app1.docx]

**LIFESTYLE AND CLINICAL SURVEY**

1. Date of Birth (mm/dd/yy) / /

2. Ethnicity (please check one)

□ Non Spanish/Hispanic/Latino

□ Mexican, Mexican American, Chicano

□ Puerto Rican

□ Cuban

□ Other Spanish/Hispanic/Latino

3. Race (please check all that apply)

□ American Indian or Alaska Native □ Vietnamese

□ Asian Indian □ Black or African American

□ Japanese □ Guamanian or Chamorro

□ Asian □ Native Hawaiian

□ Chinese □ Samoan

□ Korean □ Other Asian

□ White □ Other Pacific Islander

□ Other

4. Gender

□ Male

□ Female

5. W hat is your current marital status? (please check one)

□ Married (including common law)

□ W idowed

□ Separated

□ Divorced

□ Never married

□ Living with a significant other (not including a blood relative)

6. Please indicate the highest level of education that you completed.

□ Less than 8 years of grade school

□ Eight to 11 years of school

□ Graduated from high school

□ Some college

□ Trade school

□ Graduated from college

□ Postgraduate studies

7. Have you ever had a heart attack?

□ No (if no, please skip to #9)

□ Yes, only once

□ Yes, more than once

8. W ithin the past six months, have you had a heart attack requiring hospitalization?

□ Yes □ No

9. Have you ever had a cardiac catheterization (heart catheterization or coronary angiogram)?

□ Yes □ No

10. Have you ever had a heart bypass operation or coronary bypass surgery for blocked or clogged arteries in your heart?

□ Yes □ No

11. Have you ever had angioplasty of the coronary arteries (opening of the arteries of the heart with a balloon or other device)?

□ Yes □ No

12. Have you ever had a carotid endarterectomy or carotid angioplasty (operation for blockage or narrowing of the arteries in your neck?

□ Yes □ No

13. Have you ever had claudication or peripheral arterial disease (poor blood flow to the legs or blocked or narrowed arteries to the legs)?

□ Yes □ No

14. Have you ever had angiography (dye in the arteries of the legs) for claudication or peripheral arterial disease?

□ Yes □ No

15. Have you ever had angioplasty (balloon catheter to open blockage) in the legs?

□ Yes □ No

16. Have you ever had surgery to improve blood flow in your legs (do not include surgery for varicose veins)?

□ Yes □ No

Has a doctor ever said you had any of the following:

17. Abdominal aortic aneurysm □ Yes □ No

18. Atrial fibrillation (a type of irregular heart beat) □ Yes □ No

19. High blood pressure or hypertension (high blood) □ Yes □ No

20. High blood cholesterol □ Yes □ No

21. Congestive or chronic heart failure □ Yes □ No

22. Stroke (with continued weakness from the event) □ Yes □ No

23. Mini Stroke or TIA (symptoms of visual loss in one eye

or weakness on one side of the body that went away within a day) □ Yes □ No

24. Diabetes (sugar in the blood) □ Yes □ No

25. Kidney, eye or circulation problems because of diabetes □ Yes □ No

26. Chronic bronchitis or emphysema □ Yes □ No

27. Asthma □ Yes □ No

28. Cancer (other than squamous or basal cell skin cancer) □ Yes □ No

29. Kidney disease other than an infection or a stone □ Yes □ No

30. Stomach or duodenal ulcer □ Yes □ No

31. Ulcerative colitis or Crohn’s disease □ Yes □ No

32. Rheumatoid arthritis □ Yes □ No

33. Arthritis other than rheumatoid □ Yes □ No

34. Systemic lupus erythematosus □ Yes □ No

35. Osteoporosis (weak, thin, or brittle bones) □ Yes □ No

36. Multiple sclerosis □ Yes □ No

37. AIDS □ Yes □ No

38. Not including prolonged bed rest while being hospitalized, have you ever received blood thinners to treat or prevent blood clots?

□ No

□ Yes, only once

□ Yes, more than once

39. Have you ever had to sleep on two or more pillows to help you breathe?

□ Yes □ No

40. Have you ever been awakened at night by trouble breathing?

□ Yes □ No

41. Have you ever had swelling of your feet or ankles (excluding during pregnancy)

□ Yes □ No

42. If you answered yes to the above question, did the swelling tend to come on during the day and go down overnight?

□ Yes □ No

**The next several questions are about your menstrual history. If you are a male, please skip to question #52.**

43. Have you reached menopause? (no longer having a routine menstrual flow or period)

□ Yes □ No □ Unknown

If the answer to the above question is no or unknown, please skip the next question.

44. If yes to question #43, please check the type of menopause you experienced (otherwise, skip this question):

□ Natural

□ Radiation or Chemotherapy

□ Surgery

□ Unknown

45. Have you ever taken any kind of female hormone medication prescribed by a doctor such as

Estrogen or Progesterone for menopause?

□ Yes □ No

If you answered no to the question above, please skip to question #52.

46. W ithin the past 2 years, have you used female hormone PILLS prescribed by a doctor which contained only ESTROGEN (for example, Premarin)? (Do not include the additional use of PROGESTERONE taken along with the ESTROGEN or for a few days of the month)

□ Yes □ No □ Don’t know

47. If you answered yes to the question #46 above, for how long did you take female hormone pills containing only ESTROGEN?

□ Less than 1 month

□ 1-6 months

□ 7-10 months

□ 11-12 months

□ 13-18 months

□ 19-24 months

48. W ithin the past 2 years, have you used female hormone PILLS prescribed by a doctor which contained both ESTROGEN AND progestin (PROGESTERONE) COMBINED in the same pill or package (for example, Prempro, Premphase)?

□ Yes □ No □ Don’t know

If you answered no to question #48 above, please skip the next question.

49. In the past 2 years, how many months did use the COMBINED female hormone PILLS that contained both ESTROGEN AND PROGESTERONE in the same pill or package?

□ Less than 1 month

□ 1-6 months

□ 7-10 months

□ 11-12 months

□ 13-18 months

□ 19-24 months

50. In the past 2 years, did you use female PILLS prescribed by a doctor which contained both

ESTROGEN and TESTOSTERONE COMBINED in the same pill (for example Estratest)?

□ Yes □ No □ Don’t know

51. If you answered yes to the above question #50, how many months did you use the COMBINED

female hormone PILLS that contained both ESTROGEN AND TESTOSTERONE in the same pill?

□ Less than 1 month

□ 1-6 months

□ 7-10 months

□ 11-12 months

□ 13-18 months

□ 19-24 months

Do you currently take prescribed medication for any of the following medical problems?

| 52. Poor blood supply to the legs | □ Yes | □ | No |
| --- | --- | --- | --- |
| 53. High blood pressure | □ Yes | □ | No |
| 54. Chronic or congestive heart failure | □ Yes | □ | No |
| 55. Diabetes mellitus (high blood sugar) | □ Yes | □ | No |
| 56. Chronic bronchitis or emphysema | □ Yes | □ | No |
| 57. Asthma | □ Yes | □ | No |
| 58. Arthritis | □ Yes | □ | No |

59. Do you take any of the following medications on a regular basis?

| a. b. | Aspirin (Ecotrin, Bayer (any dose)) Ibuprofen (Motrin, Advil) | □  □ | Daily  Daily | □  □ | Weekly  Weekly | □  □ | Occasionally  Occasionally | □  □ | Never  Never |
| --- | --- | --- | --- | --- | --- | --- | --- | --- | --- |
| c. | Aleve or Naproxen | □ | Daily | □ | Weekly | □ | Occasionally | □ | Never |
| d. | BC | □ | Daily | □ | Weekly | □ | Occasionally | □ | Never |
| e. | Celebrex (Celecoxib) | □ | Daily | □ | Weekly | □ | Occasionally | □ | Never |
|  |  |  |  |  |  |  |  |  |  |
| f. | Alka Seltzer | □ | Daily | □ | Weekly | □ | Occasionally | □ | Never |

| 60. Do you now take insulin?  61. Do you now take Coumadin or Warfarin? | □  □ | Yes  Yes | □  □ | No  No |
| --- | --- | --- | --- | --- |
|  |  |  |  |  |
| 63. Do you currently take Clopidogrel (Plavix) | □ | Yes | □ | No |
| 64. Do you currently take medication for high blood cholesterol (simvastatin, Atorvastatin) | □ | Yes | □ | No |

65. Do you currently take medication for elevated levels of triglycerides or other cholesterol abnormalities (e.g., gemfibrozil, niacin, fenofibrate [Tricor])?

□ Yes □ No

66. Do you currently take Byetta or Januvia for your diabetes mellitus?

□ Yes □ No

67. Do you currently take Cilostazol (Pletal) daily to improve your walking ability?

□ Yes □ No

68. Do you currently take Trental or Pentoxifylline daily to improve your walking ability?

□ Yes □ No

69. Do you currently take any medication on a schedule other than that prescribed by your doctor? (e.g. every other day vs. daily)

□ Yes □ No

70. Are you currently taking any medications (other than the above medications) to help improve your walking ability?

□ Yes □ No (If yes, please specify)

71. Do you currently use herbal therapy or alternative medicine (acupuncture) in place of or in addition to medication prescribed by your doctor?

□ Yes □ No (If yes, please specify)

72. During your entire life, have you smoked at least 100 cigarettes?

□ Yes □ No

If the answer to the above question is no, skip to question #79.

73. How old were you when you first started smoking cigarettes regularly? (years old)

74. Do you now smoke cigarettes?

□ No (please go to the next question)

□ Yes (please skip to question #76)

75. How old were you when you quit smoking regularly? (years old)

76. On average, how many cigarettes do you usually smoke each day? (If you have stopped smoking how many cigarettes did you smoke each day?)

□ Less than 1

□ 1-4

□ 5-15

□ One pack per day

□ Greater than one pack per day

77. For how many years have you been (including your past history) a regular smoker? Do not count the times you stayed off cigarettes

□ <5 years

□ 5-9 years

□ 10-20 years

□ 21-30 years

□ 31-40 years

□ >40 years

78. Do you smoke any other form of tobacco (i.e. cigars, pipes, cigarillos)?

□ Yes □ No

79. Do you drink alcoholic beverages? □ Yes □ No

If yes to the above question, please check how often you drink alcohol:

□ Daily

□ Weekly

□ Occasionally

□ Rarely

80. Think about the walking you do outside the home. How often do you walk outside the home for more than ten minutes without stopping? (please mark only one.)

□ Rarely or never (please skip to question #83)

□ 1 time each week

□ 2-3 times each week

□ 4-6 times each week

□ 7 or more times each week

81. When you walk outside the home for more than 10 minutes without stopping, for how many minutes do you usually walk?

□ Less than 20 minutes

□ 20-39 minutes

□ 40-59 minutes

□ 1 hour or more

82. How would you describe your activity level while working (including housework)?

□ Light (sitting at a desk for more than ½ the day)

□ Moderate (frequent walking including stairs)

□ Strenuous (heavy lifting of moving of objects for at least ½ of the day)

Not including walking outside the home or work, how often each week (7 days) do you usually do the exercises below?

83. STRENUOUS OR VERY HARD EXERCISE (such as aerobic dancing, jogging, tennis, swimming laps)

□ None

□ 1 day per week

□ 2 day per week

□ 3 day per week

□ 4 day per week

□ 5 or more days per week

83a. How long do you usually exercise like this at one time?

□ Less than 20 minutes

□ 20-39 minutes

□ 40-59 minutes

□ 1 hour or more

84. MODERATE EXERCISE (biking outdoors, use of an exercise machine, easy swimming, folk dancing)

□ None

□ 1 day per week

□ 2 day per week

□ 3 day per week

□ 4 day per week

□ 5 or more days per week

85. MILD EXERCISE (slow dancing, bowling, or golf)

□ None

□ 1 day per week

□ 2 day per week

□ 3 day per week

□ 4 day per week

□ 5 or more days per week

84a. How long do you usually exercise like this at one time?

□ Less than 20 minutes

□ 20-39 minutes

□ 40-59 minutes

□ 1 hour or more

85a. How long do you usually exercise like this at one time?

□ Less than 20 minutes

□ 20-39 minutes

□ 40-59 minutes

□ 1 hour or more

86. How would you describe your current work or retirement situation? (Check one.)

□ Working at a paying job full time

□ Working at a paying job part-time

□ Retired, not working at all

□ Retired, but working part or full-time

□ Laid-off or unemployed, but looking for work

□ Laid-off or unemployed, but not looking for work

□ Not working because of disability

□ Other, please specify

87. Please specify your yearly household income by checking one of the following:

□ Less than $5,000

□ $5,000 - $30,000

□ $30,000 - $50,000

□ $50,000 - $100,000

□ > $100,000

□ Decline to Answer

□ Unknown

88. W hat language do you more commonly speak at home?

□ English

□ Spanish

□ Other, please specify
