# Supplementary material for: Use of a Smartphone App Versus Motivational Interviewing to Increase Walking Distance and Weight Loss in Overweight/Obese Adults With Peripheral Artery Disease: Pilot Randomized Trial
Source: JMIR Form Res. 2022 Feb 3;6(2):e30295. doi: 10.2196/30295 (PMC8855281; doi:10.2196/30295)
Supplement: Multimedia Appendix 2 [file formative_v6i2e30295_app2.docx]

Multimedia Appendix 2. Study outcomes by intervention groups for completers only.

|  | | **MI** |  | | | **App** | |  | | **Group Difference in Change** | |
| --- | --- | --- | --- | --- | --- | --- | --- | --- | --- | --- | --- |
| **Physiological Measures** | | **Baseline** | **Follow-up** | | | **Baseline** | | **Follow-up** | | ***P* value** | **Estimate (95% CI)** |
|  | | n = 14 | n = 14 | | | n = 11 | | n = 11 | |  |  |
|  | |  |  | | |  | |  | |  |  |
| **BMI, mean (SD)** | | 41.05 [10.17] | 40.08 [9.18] | | | 35.95 [7.88] | | 35.85 [7.90] | | 0.04 | -1.10 (-2.50, -0.10) |
| **Weight (lbs), mean**  **(SD)** | | 253.107 [59.45] | 242.14 [58.54] | | | 225.13 [58.93] | | 223.44 [59.54] | | 0.01* | -7.48 (-14.60, -3.20) |
| **Systolic Blood**  **Pressure, mean (SD)** | | 137.64 [11.69] | 134.71 [17.22] | | | 131.82 [12.63] | | 132.55 [24.93] | | 0.98 | -1.03 (-20.00, 18.00) |
| **Diastolic Blood**  **Pressure, mean (SD)** | | 80.14 [8.68] | 76.43 [10.14] | | | 75.82 [7.40] | | 75.45 [11.25] | | 0.56 | -2.00 (-12.00, 8.00) |
| **6-min Walking**  **Distance, mean (SD)** | | 260.40 [94.32] | 298.67 [101.20] | | | 326.15 [69.28] | | 331.19 [58.63] | | 0.03* | 33.60 (8.69, 58.39) |
|  | |  |  | | |  | |  | |  |  |
| **Nutritional Outcomes** | | **MI** |  | | | **App** | |  | | **Group Difference in Change** | |
|  | | **Baseline** | **Follow-up** | | | **Baseline** | | **Follow-up** | | ***P* value*** | **Estimate (95% CI )** |
| **Substitution factor,**  **mean (SD)** | | 1.95 [0.70] | 2.10 [0.84] | | | 1.84 [0.54] | | 2.22 [0.85] | | 0.57 | -0.21 (-1.08, 0.63) |
| **Modify meat factor,**  **mean (SD)** | | 2.54 [0.73] | 2.55 [0.77] | | | 2.70 [0.73] | | 2.70[0.60] | | 0.98 | 0.00(-0.67, 0.67) |
| **Avoid Frying factor,**  **mean (SD)** | | 3.26 [0.59] | 3.30 [0.54] | | | 3.09 [0.63] | | 3.37 [0.48] | | 0.54 | -0.08 (-0.58, 0.25) |
| **Replacement factor,**  **mean (SD)** | | 1.95 [0.72] | 2.31 [0.81] | | | 2.18 [0.48] | | 2.36 [0.63] | | 0.47 | 0.33(-0.33, 0.83) |
| **Avoid Fat factor,**  **mean (SD)** | | 2.26 [0.74] | 1.99 [0.74] | | | 2.20 [0.58] | | 2.39 [0.65] | | 0.24 | -0.42(-1.00, 0.25) |
| **Total dietary factors** | | 2.40 [0.47] | 2.45 [0.52] | | | 2.40 [0.21] | | 2.61 [0.32] | | 0.62 | -0.11(-0.53 0.29) |
|  | |  |  | | |  | |  | |  |  |
| **Quality of Life Measures** | | **MI** | | | | **App** | | | | **Group Difference in Change** | |
|  | | **Baseline** | **Follow-up** | | | **Baseline** | | **Follow-up** | | ***P* value*** | **Estimate(95% CI)** |
|  | |  |  | | |  | |  | |  |  |
| **VascuQol Score, mean (SD)** | 4.28 [0.91] | | |  | 5.36 [0.75] | | 4.42 [1.18] | | 5.09 [1.37] | 0.18 | 0.48(-0.20, 1.13) |
| **Activity Score, mean**  **(SD)** | | 3.78 [0.95] | 4.96 [0.89] | | | 3.77 [1.41] | | 4.53 [1.49] | | 0.24 | 0.38 (-0.29, 1.12) |
| **Symptom Score,**  **mean (SD)** | | 4.07 [1.37] | 5.21 [0.90] | | | 4.70 [1.45] | | 5.30 [1.35] | | 0.23 | 0.75 (-0.75, 1.75) |
| **Pain Score** | | 3.71 [1.21] | 4.91 [1.04] | | | 4.00 [0.99] | | 4.82 [1.38] | | 0.49 | 0.50 (-0.75, 1.25) |
| **Emotional Score,**  **mean (SD)** | | 4.79 [1.09] | 5.85 [0.83] | | | 4.87 [1.48] | | 5.49 [1.62] | | 0.12 | 0.62 (-0.29, 1.43) |
| **Social Score, mean**  **(SD)** | | 4.86 [1.67] | 5.82 [1.10] | | | 5.00 [1.52] | | 5.59 [1.96] | | 0.47 | 0.50 (-1.00, 2.00) |
| **Exercise Measure** | | **MI** | | | | **App** | | | | **Group Difference in Change** | |
|  | | **Baseline** | **Follow-up** | | | **Baseline** | | **Follow-up** | | ***P* value*** | **Estimate (95% CI)** |
| **Exercise Score (min)** | | 14.11 [18.67] | 27.32[27.18] | | | 16.14[19.15] | | 22.73[16.56] | | 0.62 | 2.50 (-7.50, 17.50) |

^*^Significance of change from baseline to 3 months between the MI and App group. *BMI: Body Mass Index; VascuQol: Vascular Quality of Life Questionnaire Score*
